# Identification of *Pratylenchus coffeae* as a causal agent of root rot disease in *Sorghum bicolor* in China

**DOI:** 10.1186/s12866-025-03759-1

**Published:** 2025-01-24

**Authors:** Ling Qin, Fan-kang Lin, Yun-long Lv, Ze-Lin Tai, Xu Zhang, Hong-lian Li, Yu Li, Ke Wang

**Affiliations:** 1 College of Plant Protection, Henan Agricultural University, Zhengzhou, Henan Province 450046 P. R. China; 2 National Key Laboratory of Wheat and Maize Crop Science, Henan Agricultural University, Zhengzhou, Henan Province 450046 P. R. China

**Keywords:** *Sorghum bicolor*, Root-lesion nematode, Identification, Parasitism, Pathogenicity

## Abstract

Sorghum (*Sorghum bicolor*) is an important food and feed crop. Root-lesion nematodes (*Pratylenchus* spp.) are a group of pathogenic nematodes that cause severe economic losses in various food and cash crops. This study identified diseased sorghum plants with stunted growth and brown, rotting roots in sorghum fields in Shanxi Province, China. A species of root-lesion nematode was isolated by modified Baermann funnel method and named the GL-1 population. Afterward, the GL-1 population of root-lesion nematodes was identified as *P. coffeae* through a combination of morphological, rDNA-ITS and rDNA-28 S D2-D3 region techniques for molecular biological identification. We also conducted greenhouse experiments to assess the parasitism and pathogenicity of GL-1 and four other *P. coffeae* populations on sorghum through pot inoculation. At 60 days after inoculation, the results indicated that all five populations of *P. coffeae* were capable of infecting and causing damage to the sorghum plants. Sorghum is a suitable host for *P. coffeae* (with a reproduction factor > 1). Moreover, compared with those in the control group, the aboveground fresh weights and root fresh weights of sorghum in the five inoculation groups were significantly lower, and brown spots or even necrotic rot appeared on the roots. All five populations were highly pathogenic to sorghum, but there were significant differences in pathogenicity among the populations. This study provides a scientific basis for identifying and detecting root-lesion nematodes in sorghum.

**Supplementary Information:**

The online version contains supplementary material available at 10.1186/s12866-025-03759-1.

## Introduction

Sorghum (*Sorghum bicolor*), a member of the Gramineae family in the Sorghum genus, originated from Ethiopia, in Africa [1]. Sorghum is not only the world’s fifth-most important food and feed crop, but it is also used in the energy and processing industries [2]. As of 2022, sorghum is grown on 7.29 × 10^5^ hectare in China, with production reaching 3.32 × 10^8^ t (https://data.stats.gov.cn/easyquery.htm?cn=C01). China’s sorghum is primarily used in the production of liquor, including the world-famous ‘China-Moutai’, ‘China-Wuliangye’, etc., which are brewed with sorghum as the main raw material, supporting a brewing industry with distinct Chinese characteristics [3].

Root-lesion nematodes (*Pratylenchus* spp.) are a very important group of migratory endoparasitic nematodes with a wide host range that, together with cyst nematodes and root-knot nematodes, are among the most damaging plant pathogenic nematodes worldwide [4]. The symptoms of root-lesion nematode damage are easily confused with those caused by a lack of water and fertilizer, soil-borne fungal diseases, and environmental stress [5]. Moreover, the mechanical damage caused by root-lesion nematodes when they infect plant roots provides more favorable conditions for infecting pathogens such as fungi and bacteria [6, 7]. Among the sorghum nematode diseases, three nematodes are more serious, namely, *Belonolaimus* spp., *Meloidogyne* spp. and *Pratylenchus* spp [8–10]. These nematode diseases can severely impair sorghum production, leading to substantial reductions in both yield and quality [11]. Consequently, it is imperative to implement effective monitoring and control strategies to manage sorghum nematode diseases efficiently.

In 2021, we detected weak growth and root rot symptoms in some sorghum plants from Shanxi Province, China. A large number of root-lesion nematodes were isolated using a modified Baermann funnel method [12], and the root-lesion nematodes collected in Shanxi Province were clearly identified as *P. coffeae* by morphological and molecular biology methods. Inoculation of greenhouse pots, based on the nematode host plant determination criteria proposed by Goo et al. [13] confirmed that sorghum is a suitable host for *P. coffeae* and that *P. coffeae* is highly pathogenic to sorghum. Koch’s law experiments verified that the collected *P. coffeae* was the pathogen causing sorghum root rot in this study. Additionally, the parasitism and pathogenicity of five different populations of *P. coffeae* on sorghum and the differences in pathogenicity between populations were determined. In this study, sorghum root rot nematode disease caused by *P. coffeae*, was found for the first time in Shanxi Province, China. This is the first time that differences in the pathogenicity of different *P. coffeae* populations on sorghum have been clarified. This study provides a scientific basis for research related to the detection and integrated control of root rot in sorghum.

## Materials and methods

### Isolation and purification of nematodes

Diseased sorghum (Jinza 210) with dwarfed plants, reduced root systems and brown rot was found in a sorghum field in Yangfang village, Mengfeng township, Qingxu county, Taiyuan, Shanxi Province, China, and samples of the sorghum rhyzosphere soil were subsequently collected. Root tissues from diseased plants were cut and mixed with inter-root soil, and root-lesion nematodes were isolated from the samples using a modified Baermann funnel method [12]. Single female root-lesion nematodes were picked under a stereomicroscope, disinfected with 0.3% streptomycin sulfate solution about 6 h [14], washed with sterile water, inoculated onto carrot disks, and cultured in the dark at 25 ℃ for approximately 90 days to obtain a purified population of root-lesion nematodes [15], which were named the GL-1 population.

### Morphological identification of root-lesion nematodes

Purely cultured root-lesion nematodes were picked onto concave slides containing sterile water under a stereomicroscope; subsequently, the nematodes were heat-killed, fixed in FG solution (formalin: Glycerin: water = 10 : 1 : 89) [16], and dehydrated by glycerol-ethanol dehydration. Lastly, a permanent microscopic mount was made by wax circle method [12]. The nematodes were measured and photographed with a Nikon Eclipse TI-S inverted microscope (Japan). The measurement and calculation of quantitative characteristics are based on deMan formula and Hooper et al. [12].

### Molecular identification of root-lesion nematodes

The DNA of single nematodes was extracted by protease K method [17]. The internal transcribed spacer region (ITS) rDNA gene was amplified using primers TW81 (5’-GTTTCCGTAGGTGAACCTGC-3’) and AB28 (5’-ATATGCTTAAGTTCAGCGGGT-3’) [18]. The D2-D3 region of the 28 S rDNA gene was amplified using primers D2A (5’-ACAAGTACCGTGAGGGAAAGTTG-3’) and D3B (5’-TCGGAAGGAACCAGCTACTA-3’) [19]. The reaction system was formulated according to the instructions of 2×Magic Green Taq SuperMix Polymerase (TOLOBIO, Shanghai, China) for PCR. The reaction procedure was as follows: pre-denaturation at 95 ℃ for 3 min, followed by 35 cycles of denaturation at 95 ℃ for 30 s, annealing at 59.1 ℃ (ITS rDNA) or 51.9 ℃ (28 S rDNA) for 30 s, extension at 72 ℃ for 2 min, and a final extension at 72 ℃ for 5 min. The resulting PCR products were purified with a FastPureⓇ Gel DNA Extraction Mini Kit (Vazyme Biotech Co., Ltd., Nanjing, China), connected to a one-step ZTOPO-Blunt/TA cloning vector (Zoman, Beijing, China), transferred to DH5α cells (TOLOBIO), and then sent to Sangon Biotech Co., Ltd. (Shanghai, China) for sequencing [15].

### Phylogenetic relationship analysis of the root-lesion nematodes

The obtained sequences of the rDNA-ITS and rDNA 28 S D2-D3 regions of the GL-1 population were submitted to the GenBank database with the accession numbers PP531525 (rDNA-ITS) and PP531576 (28s rDNA), respectively, and then the corresponding gene sequences of *Pratylenchus* obtained from the GenBank database were aligned using the BLASTn algorithm (http://blast.ncbi.nlm.nih.gov/Blast.cgi). Multiple sequence comparison was performed in MEGA 7 using the ClustalW technique. The optimal nucleotide substitution model GTR + G + I was selected under the Akaike information criterion (AIC) using PAUP and MrModeltest 2.3, and a phylogenetic tree of 28 S and ITS sequences in *P. coffeae* was constructed using MrBayes 3.1 [20].

### Parasitism and pathogenicity of five *P. coffeae* populations toward *Sorghum bicolor*

In this study, the parasitism and pathogenicity of four other populations of *P. coffeae* conserved in our laboratory and GL-1 population (Table 1) in sorghum were determined via pot inoculation experiments. Sorghum seeds were sterilized with 75% ethanol for 3–5 min, washed with sterile water and sown in sterile soil for 15 days of growth. The flowerpots used in the experiment were the same, with an outer diameter of 20 cm, an inner diameter of 17.5 cm, a bottom diameter of 13.5 cm and a height of 18 cm, which could hold a volume of about 3.5 L of nutrient soil. Subsequently, sorghum seedlings with uniform growth were selected and inoculated with a suspension of five *P. coffeae* populations at a concentration of 1000 nematodes/mL around the sorghum root system at a rate of 2000 nematodes/pot. The nematode suspension contains all the life stages of the nematodes, i.e., eggs, females, males, and juveniles. Five biological replicates were performed. The sorghum treatment without nematode inoculation was used as a control. All the pot experiments were replicated twice in the greenhouse (28 ℃, 12 h light/12 h dark photoperiod). The potted seedlings were watered 1 day before inoculation with nematodes to moisten the soil, and the pots were not watered for 3 days after inoculation to ensure normal nematode infestation; then regular planting management was applied [21]. Sixty days after inoculation with nematodes, growth parameters such as the plant height, aboveground fresh weight and root fresh weight of sorghum were investigated, and symptoms of sorghum root infestation were observed and imaged [16]. The modified Baermann funnel method [12] was used to isolate and count the number of nematodes (Pf) in the sorghum inter-root region and to calculate the reproduction factor (Rf) of the nematodes. The host plant determination criteria proposed by Goo et al. [13] were used to determine whether sorghum was a host plant for *P. coffeae* during this test.

**Table 1:** Nematode populations used in this study

Nematode population	Host	Sampling location	In vitro cultured	GenBank (ITS)	GenBank (28 S)
HN-K1	corn	Pingdingshan City, Henan Province	Carrot disks	OR417384	OR417283
SD-YC-1	tobacco	Weifang City, Shandong Province	Carrot disks	OQ449389	OQ449390
HN-A21	sesame	Xuchang City, Henan Province	Carrot disks	MN588280	MN588282
XC-344-1	soybean	Linyi City, Shandong Province	Carrot disks	MT879294	MT879295
GL-1	sorghum	Taiyuan City, Shanxi Province	Carrot disks	PP531525	PP531576

### Histopathological observation

The *P. coffeae* in the root system of the differently treated sorghum plants were stained with acidic magenta and observed according to the methods by Bybd et al. [22]. Tissue sectioning of the sorghum root system was performed after dehydration, transparency, wax dipping, embedding, sectioning, staining and sealing according to the methods of Wang et al. [23] and Sasanelli et al. [24]. The sections were stained with Fanshi red-solid green dye and then observed and photographed under a microscope.

### Data analysis

Statistical analysis was performed with SPSS 22.0 software (Chicago, USA) in this study. All the data were subjected to a one-way analysis of variance (ANOVA) and tested for differences among treatments at the 5% level by Duncan’s multiple range test (DMRT) [14].

## Results

### Field symptoms

**Fig. 1:**
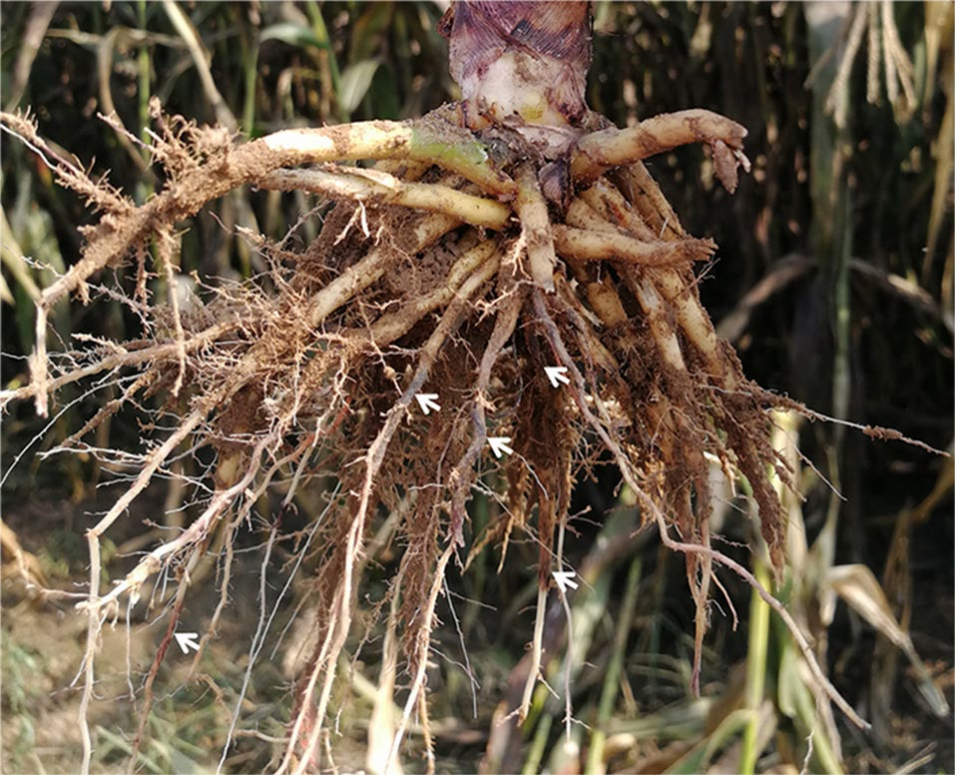
Root rot field symptoms of sorghum caused by root-lesion nematodes in Shanxi Province, China

As a result of root-lesion nematode infestation, the symptoms in the sorghum field were as follows: the aboveground plants were significantly shorter and weaker, the root systems were reduced, the roots showed obvious brown or black-brown spots, and parts of the root systems showed varying degrees of necrosis and decay (Fig. 1).

### Morphological identification of *P. coffeae*

**Fig. 2:**
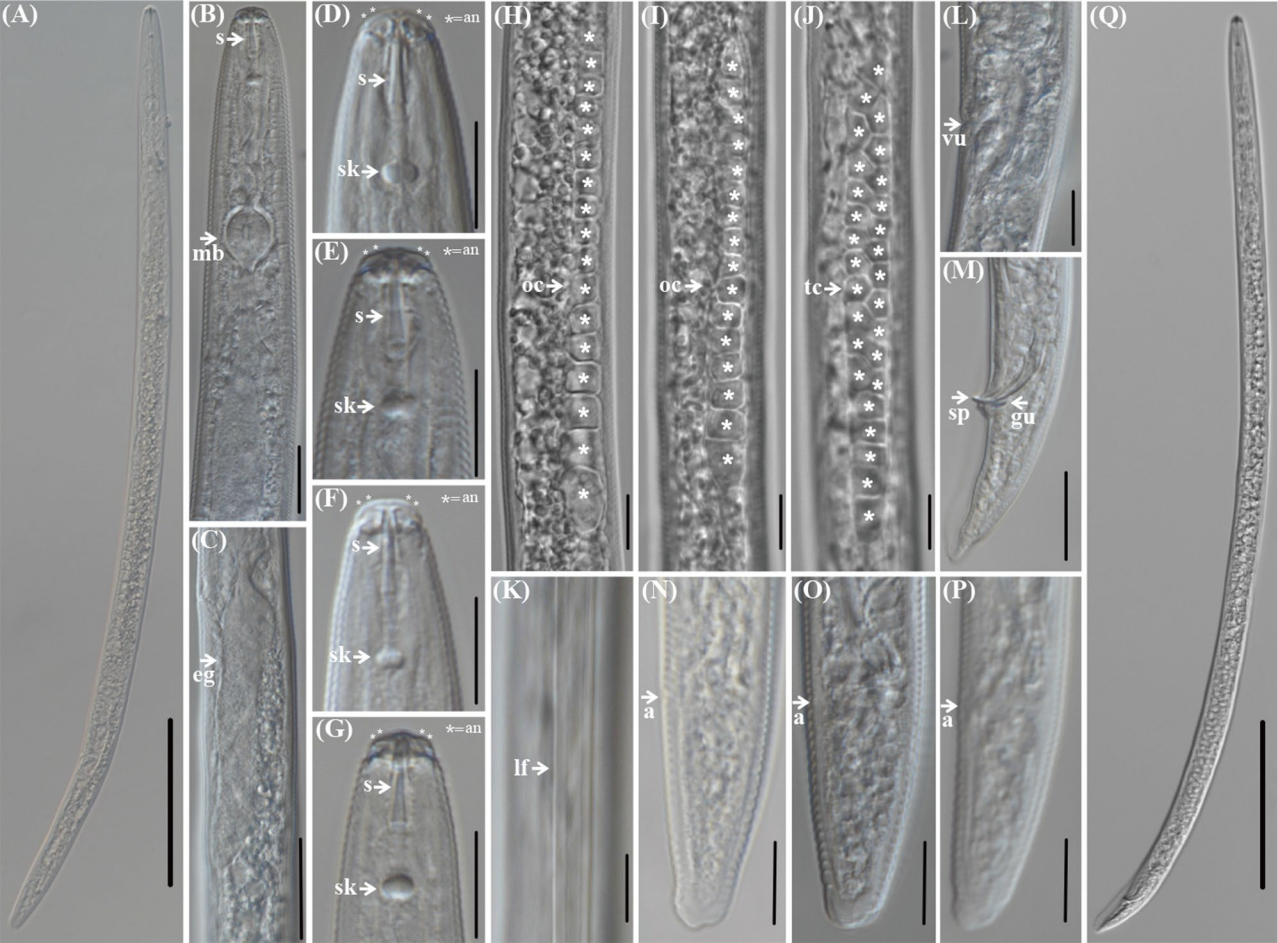
Light micrographs of *Pratylenchus coffeae* from sorghum in Shanxi Province, China. (**A**) female entire body; (**B**) anterior region; (**C**) pharyngeal gland lobe overlapping the intestine; (**D**)-(**G**) lip region; (**H**)-(**I**) female anterior end of genital gland; (**J**) male anterior end of genital gland; (**K**) lateral field; (**L**) post-vulval region showing the post-uterine sac; (**M**) male tail region; (**N**)-(**P**) female tail region; (**Q**) male entire body. Scale bars: 100 μm ((**A**), (**Q**)) and 10 μm ((**B**)-(**P**)). S, stylet; mb, median bulb; e.g., esophageal glands; sk, stylet knob; an, annuli; oc, ovary cells; lf, lateral field; a, anal; tc, testis cells; vu, vulval; sp, spicules; gu, gubernaculum

Morphological characteristics: The morphometric values for the female and male root-lesion nematodes harvested from the sorghum inter-roots in Shanxi Province, China were compared with the corresponding indices of other *P. coffeae* nematodes, as shown in Table 2; the microscopic observations of their morphometric features are shown in Fig. 2.

**Table 2:** Morphometrics of a population of *Pratylenchus coffeae* collected in Taiyuan City, Shanxi Province, China

Character	GL-1 population			Pratylenchus coffeae population of Inserra et al. [25]	
	female	male		female	male
n	18	18		20	20
l	583.55 ± 30.47 (514.35–644.50)	536.58 ± 44.19 (482.90-641.80)		601.90 ± 51.40 (520.00-715.00)	589.10 ± 25.40 (558.50-647.50)
a	22.52 ± 2.62 (18.92–28.15)	28.85 ± 1.70 (26.29-32.00)		28.70 ± 3.10 (23.40–34.00)	30.80 ± 1.60 (28.70–33.90)
b	5.67 ± 0.20 (5.36–6.24)	5.86 ± 0.25 (5.25–6.20)		6.70 ± 0.40 (5.60–7.20)	6.70 ± 0.30 (6.00-7.30)
b’	4.81 ± 0.32 (4.02–5.26)	-		-	-
c	20.76 ± 2.38 (18.05–27.99)	21.18 ± 2.02 (18.12–26.70)		20.90 ± 2.80 (17.00–31.00)	21.80 ± 1.60 (19.40–25.40)
c’	1.91 ± 0.23 (1.52–2.38)	-		-	-
V	78.90 ± 1.70 (75.56–82.40)	-		80.50 ± 1.50 (76.00-82.50)	-
T	-	39.80 ± 5.51 (31.17–52.19)		-	-
DGO form stylet base	3.18 ± 0.42 (2.18–3.81)	3.37 ± 0.48 (2.35–3.94)		2.80 ± 0.50 (2.50-4.00)	3.60 ± 0.30 (3.00–4.00)
EP	87.40 ± 4.09 (79.21–94.07)	85.42 ± 4.37 (78.92–94.19)		-	-
Stylet length	16.23 ± 0.53 (15.36–17.29)	16.00 ± 0.72 (14.37–16.91)		16.90 ± 0.20 (16.50–17.00)	15.00 ± 0.40 (14.50–15.50)
Stylet shaft	10.59 ± 1.81 (7.04–13.21)	10.45 ± 1.65 (8.29–13.91)		-	-
Stylet knob height	2.55 ± 0.51 (1.68–3.41)	2.43 ± 0.47 (1.70–3.07)		-	-
Stylet knob width	3.86 ± 0.38 (3.19–4.54)	3.35 ± 0.54 (2.25–4.17)		-	-
Pharyngeal overlap	39.66 ± 8.05 (25.17–53.21)	29.33 ± 5.27 (24.31–46.90)		49.80 ± 10.30 (34.00-72.50)	-
Max body width	26.29 ± 3.78 (21.53–34.07)	18.70 ± 2.25 (15.09–23.98)		-	-
Tail length	28.46 ± 3.67 (22.05–35.70)	25.67 ± 4.21 (19.06–35.42)		29.00 ± 3.30 (21.50–36.00)	27.10 ± 1.90 (24.50–31.00)
Number of tail annuli	24.17 ± 1.58 (18–26)	-		-	-
Vulva to anus distance	86.86 ± 6.36 (76.10-99.24)	-		87.20 ± 12.80 (70.50–135.00)	-
Post-uterine sac length	27.70 ± 4.3 (18.21–35.20)	-		29.50 ± 6.50 (19.50–49.50)	-
Lateral field width	7.36 ± 0.57 (6.53–8.74)	5.90 ± 0.85 (4.15–8.53)		-	-
Vulval body diameter	19.13 ± 1.47 (16.72–22.07)	-		-	-
Anal body diameter	17.99 ± 2.94 (12.79–23.15)	-		-	-
Spicule length	-	17.10 ± 0.73 (15.41–18.29)		-	17.50 ± 0.60 (16.00–18.00)
Gubernaculum length	-	5.20 ± 0.3 (4.68–5.79)		-	5.30 ± 0.30 (5.00-5.50)

Notes: All measurements are in µm and in the form of mean ± standard deviation (range). n, number of specimens measured; l, body length; a, body length/greatest body width; b, body length/length from the lips to the junction of oesophageal gland and intestine; b’, body length/length from the lips to oesophageal gland end; c, body length/tail length; c’, tail length/tail diameter at anus; V, distance of vulva from the lips × 100/body length; T, distance form cloaca opening to anterior most part of testis/body length × 100%; DGO, distance between dorsal oesophageal gland opening and stylet knobs

Females almost straight, when heat-killed (Fig. 2A). Head sclerotization heavy, extending backward. The labial region is low and slightly offset, containing 2 labial rings (Fig. 2D-G). The median bulb is ovoid (Fig. 2B) and the esophageal glands cover the anterior end of the intestine (Fig. 2C). Lateral fields with distinct four lines at mid-body (Fig. 2K). The oocytes in the ovary were arranged in a single row, and the spermatheca was distinct and round or oval (Fig. 2H-I). Tail subcylindrical, caudal end occasionally with distinctly irregular indentations (Fig. 2N-P). The morphology of the male was similar to that of the females after heat exposure (Fig. 2Q). The seminal vesicle is obvious (Fig. 2J). Male tail with slender spicules, narrowing to a pointed terminus (Fig. 2M).

The morphological characteristics of the root-lesion nematode population collected in this study were more consistent with those of *P. coffeae* in the article by Inserra et al. [25] and Xie [26], so the root-lesion nematode population collected from the inter-roots of sorghum from Shanxi Province, China, was tentatively identified as *P. coffeae.*

### Molecular identification and phylogenetic analysis of *P. coffeae*

The rDNA-ITS and rDNA-28 S D2-D3 regions of the sorghum inter-root nematode from Shanxi Province, China were PCR-amplified and sequenced using the universal primers TW81/AB28 and D2A/D3B, and two sequences with lengths of 1086 bp and 811 bp, respectively, were obtained. A BLAST analysis of the NCBI database showed that the sequence of the rDNA-ITS region from the root-lesion nematode obtained in this study (GenBank accession no. PP531525) was 100% similar to the rDNA-ITS region sequence of the root-lesion nematode *P. coffeae* (OQ592785, OQ592735). The sequence of the rDNA-28 S D2-D3 region (GenBank accession no. PP531576) showed 100% similarity to the sequence of the rDNA-28 S D2-D3 region of the root-lesion nematode *P. coffeae* (OQ592691, OQ592693).

**Fig. 3:**
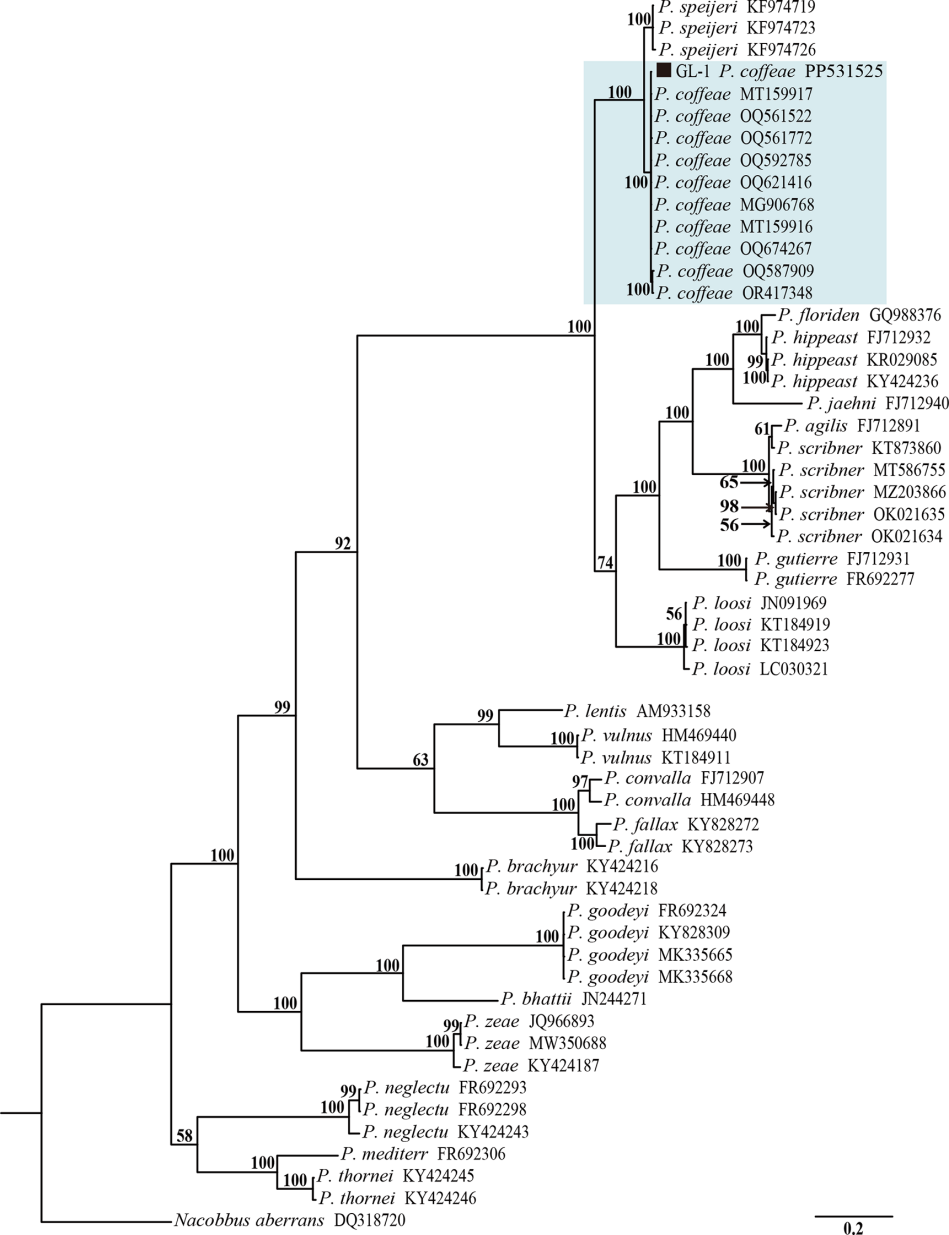
Bayesian tree of *Pratylenchus* inferred from ITS rRNA gene sequences under the GTR + I + G model. Posterior probabilities of greater than 50% are given for appropriate clades. The newly obtained sequence is indicated with a black square

**Fig. 4:**
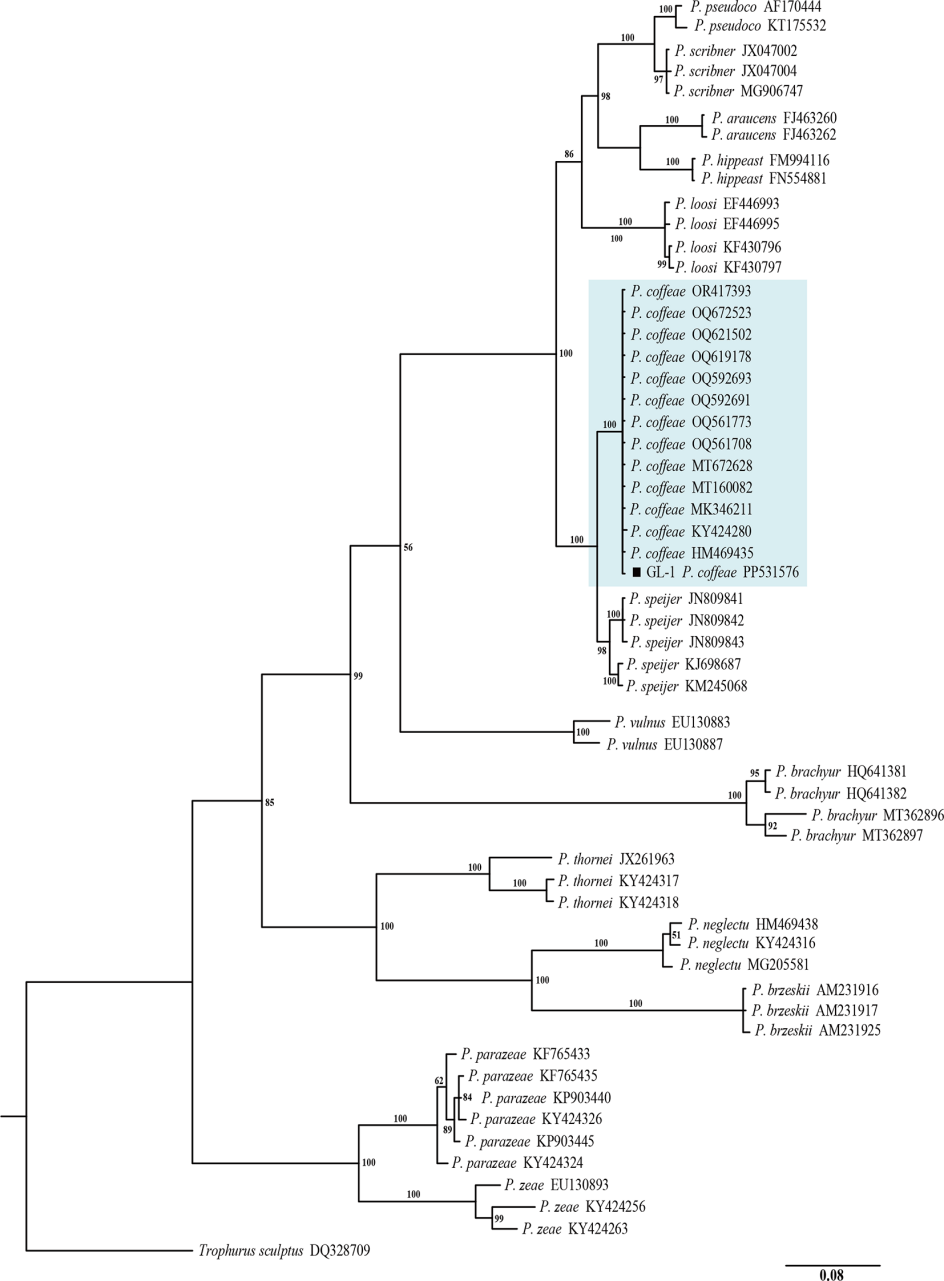
Bayesian tree of *Pratylenchus* inferred from 28 S rRNA gene sequences under the GTR + I + G model. Posterior probabilities of greater than 50% are given for appropriate clades. The newly obtained sequence is indicated with a black square

The Bayesian phylogenetic tree constructed based on the sequences of the rDNA-ITS region contained one outgroup and 53 ingroups based on the *Nacobbus aberrans* population (Fig. 3), and the sequences of the root-lesion nematodes isolated from the sorghum root system in Shanxi Province clustered in the same highly supported branch as the reported *P. coffeae* rDNA-ITS region with a support rate of up to 100%. The Bayesian phylogenetic tree constructed based on the sequence of the rDNA-28 S D2-D3 region contained one outgroup and 55 ingroups based on the *Trophurus sculptus* population (Fig. 4), and this root-lesion nematode sequence was clustered in the same highly supported branch as the reported *P. coffeae* rDNA-28 S D2-D3 region, with up to 100% similarity. The above molecular identification results based on the sequences of the rDNA-ITS and rDNA-28 S D2-D3 regions were consistent with the morphological findings, indicating that the root-lesion nematode GL-1 population isolated from sorghum in Shanxi Province, China was *P. coffeae*.

### Parasitism and pathogenicity assays of five *P. coffeae* populations from *Sorghum bicolor*

**Fig. 5:**
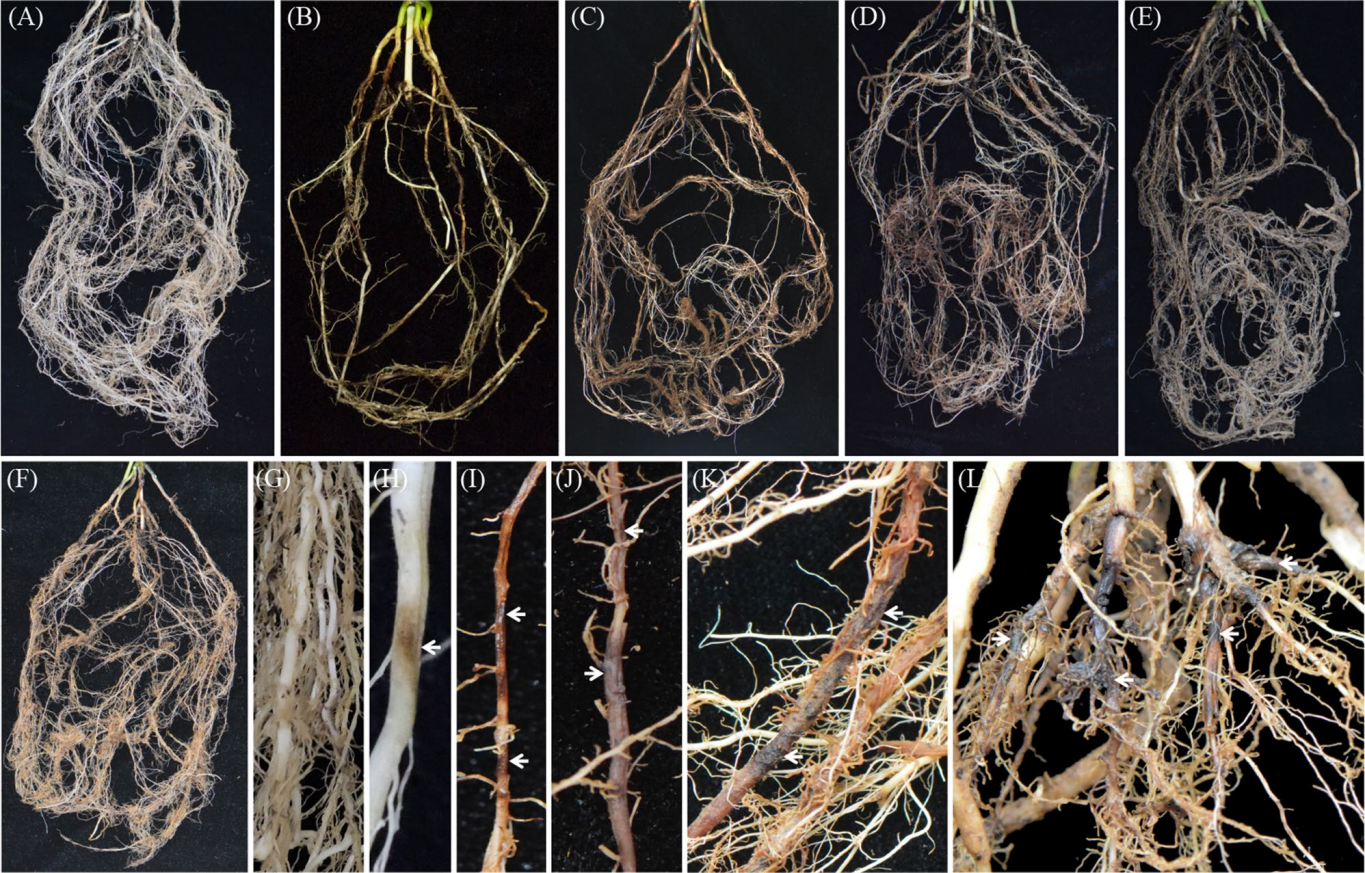
Five populations of *Pratylenchus coffeae* suppressed the growth of *Sorghum bicolor* roots. (**A**) Root system of a non-inoculated plant; (**B**)-(**F**) roots of plants 60 days after inoculation with the HN-K1, SD-YC-1, HN-A21, XC-344-1 and GL-1 populations, respectively; (**G**) healthy roots; and (**H**)-(**L**) necrotic areas on the root systems from *Sorghum bicolor* inoculated with *P. coffeae*

The results of potting at 60 days after inoculation showed (Fig. 5A-F) that the root systems of the plants in the treatment groups inoculated with five different populations of *P. coffeae* showed different levels of damage. Compared with control plants not inoculated with the nematode, sorghum plants inoculated with *P. coffeae* exhibited slow aboveground growth and significantly shorter and weaker plants compared to control plants not inoculated with the nematode. As a result of *P. coffeae* infestation, the root system of sorghum plants is significantly reduced, the roots tend to break off, the fibrous roots are reduced, and some of the roots develop brown or dark brown spots. In the early stages of *P. coffeae* infestation, small, light brown or tan spots appeared in the sorghum root system (Fig. 5H), and as the disease continued to worsen and the nematodes continued to feed and reproduce in the root system, the spots in the sorghum root system continued to expand (Fig. 5I-K), ultimately leading to large, necrotic rot-like conditions throughout the root system (Fig. 5L). The roots of the control sorghum without nematode connections grew healthily and showed no symptoms of damage (Fig. 5G).

Sixty days after inoculation, large numbers of *P. coffeae* were isolated from sorghum roots and inter-root soil (Table 3). Among them, sorghum inter-root soils inoculated with the HN-K1 and SD-YC-1 populations had the highest reproduction capacity, with averages of 4971 and 4632 nematodes and Rf values of 2.5 and 2.3, respectively. The Rf values of *P. coffeae* in the inter-root regions of sorghum plants inoculated with the HN-A21, XC-344-1 and GL-1 populations were 2.2, 2.1 and 1.6, respectively. Based on Goo’s host determination criteria [13], all five populations of *P. coffeae* inoculates were capable of infesting and damaging sorghum and the sorghum was a suitable host for *P. coffeae* (Rf > 1), but there were differences in the fecundity of different sorghum populations.

**Table 3:** Final population densities (pf) and reproduction factor (rf) of five populations of *Pratylenchus coffeae* on *Sorghum bicolor* 60 days after the inoculation of 2000 nematodes/pot

Treatment population	Nematodes in the soil	Nematodes in the root	Total nematodes	Rf
CK	0	0	0	0
HN-K1	4038.0 ± 1226.1^a^	595.0 ± 86.8^a^	4633.0 ± 1252.9^a^	2.3 ± 0.6^a^
SD-YC-1	4358.0 ± 1460.0^a^	613.0 ± 215.4^a^	4971.0 ± 1670.1^a^	2.5 ± 0.8^a^
HN-A21	3936.8 ± 1891.8^a^	460.8 ± 192.7^ab^	4397.6 ± 2080.4^a^	2.2 ± 1.0^a^
XC-344-1	3792.6 ± 1137.9^ab^	483.4 ± 85.3^a^	4276.0 ± 1201.5^a^	2.1 ± 0.6^a^
GL-1	2907.6 ± 1019.2^b^	306.8 ± 87.1^b^	3214.4 ± 1080.4^b^	1.6 ± 0.5^b^

Note: Data are mean ± standard error of five replicates. Different lowercase letters within the same columns indicate significant difference at 0.05 level. The same below. Reproductive factor (Rf ) = final isolated nematodes/initial inoculation nematodes

Sorghum plants from five different populations inoculated with *P. coffeae* as described above were surveyed to measure the plant height, aboveground fresh weight and root fresh weight to observe their pathogenicity (Table 4). The average aboveground plant height, aboveground fresh weight and root fresh weight of uninoculated healthy sorghum plants were 158.8 cm, 40.1 g and 36.5 g, respectively. Sorghum plants inoculated with the HN-K1 and SD-YC-1 populations had significantly shorter plants and more severely damaged root systems, with plant heights, aboveground fresh weights and root fresh weights of 71.3 cm, 21.2 g, and 11.7 g and 69.9 cm, 21.8 g, and 12.6 g, respectively, which were significantly lower (*P* < 0.05) than those of the uninoculated control. Sorghum plants inoculated with the GL-1 population exhibited relatively little plant growth and relatively severe root damage, with an average plant height, aboveground fresh weight, and root fresh weight of 102.4 cm, 30.3 g, and 20.3 g, respectively. In summary, the five different populations of *P. coffeae* tested here were all clearly pathogenic to sorghum, but there were significant differences in the pathogenicity of the different populations, with the strongest pathogenicity to sorghum being found in the HN-K1 and SD-YC-1 populations, followed by the HN-A21 and XC-344-1 populations, and the weakest pathogenicity to sorghum in the GL-1 population, which was isolated from Shanxi Province, China.

**Table 4:** Effects of five different populations of *Pratylenchus coffeae* on the growth of *Sorghum bicolor* 60 days the inoculation 2000 nematodes/pot

Treatment population	Plant height (cm)	Fresh shoot weight (g)	Fresh root weight (g)
CK	158.8 ± 17.7^a^	40.1 ± 11.3^a^	36.5 ± 5.0^a^
HN-K1	71.3 ± 4.3^c^	21.2 ± 3.5^c^	11.7 ± 1.2^c^
SD-YC-1	69.9 ± 10.2^c^	21.8 ± 9.8^bc^	12.6 ± 5.2^c^
HN-A21	78.2 ± 13.9^bc^	21.7 ± 4.3^bc^	14.9 ± 5.6^bc^
XC-344-1	83.1 ± 8.0^bc^	24.4 ± 11.5^bc^	17.0 ± 6.6^bc^
GL-1	102.4 ± 32.1^b^	30.3 ± 9.9^b^	20.3 ± 3.0^b^

Note: Data are mean ± standard error of five replicates. Different lowercase letters within the same columns indicate significant difference at 0.05 level. The same below

### Histopathological observation

**Fig. 6:**
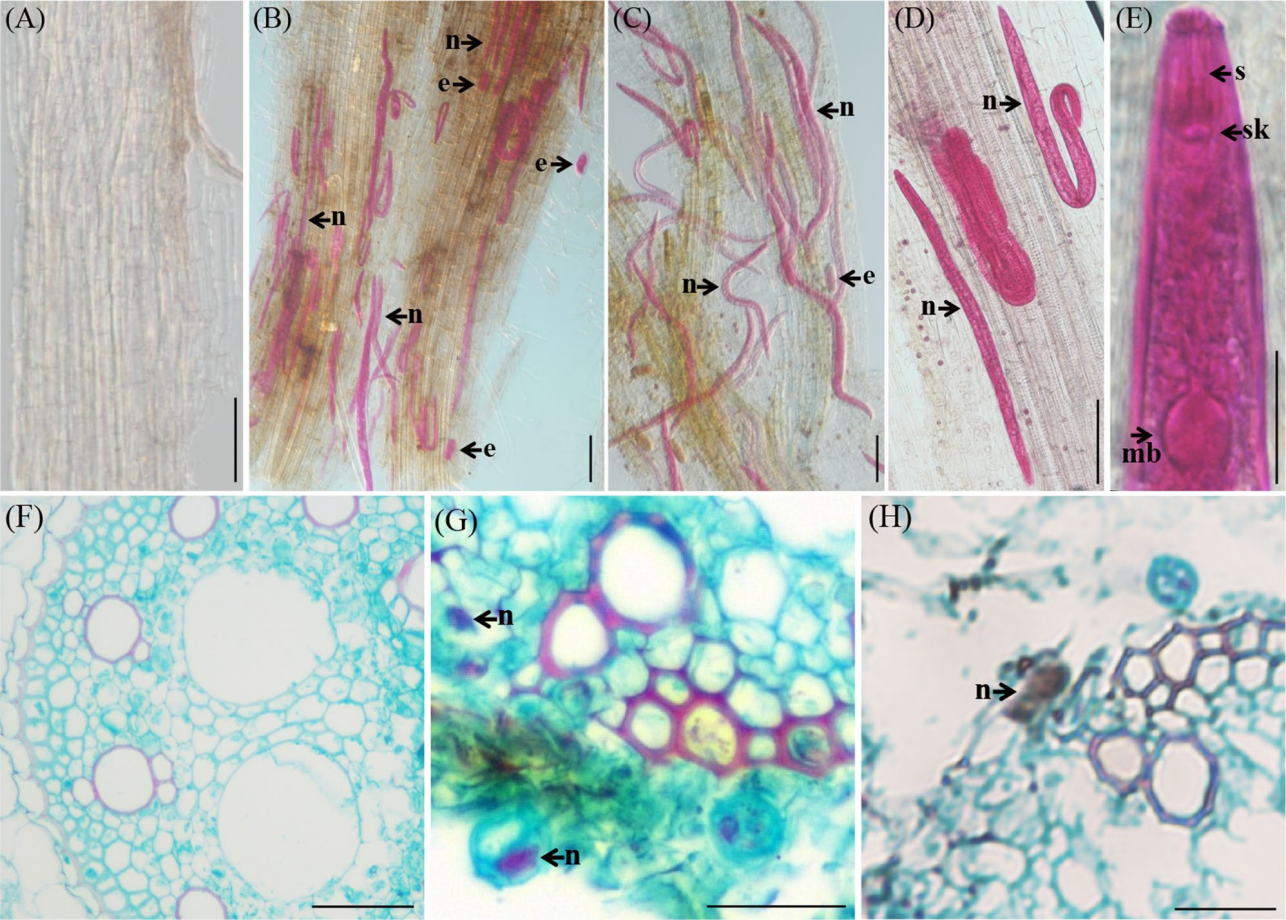
*Sorghum bicolor* roots infected by *Pratylenchus coffeae*. (**A**) Healthy roots; (**B**)-(**E**) a large number of *P. coffeae* individuals and eggs in the cortex of the roots; (**F**) *P. coffeae* in Sorghum root cells; (**G**) cross section of undamaged roots; and (**H**)-(**J**) cross section of roots infected with *P. coffeae* showing vermiform nematodes in damaged epidermal cells. Scale bars: 100 μm (**A**)-(**E**); 20 μm (**F**); 50 μm (**G**)-(**H**). n, nematode; e, egg; s, stylet; sk, stylet knob; mb, median bulb

After acidic magenta staining of the morbid sorghum root system, a large number of *P. coffeae* individuals and eggs were observed within the roots under the microscope, indicating that the nematode can grow, develop and complete its life cycle within the sorghum root system of the test (Fig. 6B-E). The results of the pathologic tissue sections showed (Fig. 6G-H) that *P. coffeae* was present in the cells of sorghum roots, and due to the migration and feeding of this nematode, the cells of the root tissues were partially degraded, which in turn led to the decay and necrosis of the roots.

## Discussion

In this study, we used a combination of morphological and molecular biological identification of root-lesion nematode species harvested from the inter-roots of sorghum in Shanxi Province, China. The results indicated that this nematode population was basically consistent with the morphological characteristics of *P. coffeae* as previously reported. Phylogenetic trees also indicate that this nematode population is in the same branch as *P. coffeae* and is distinguished from other populations of the genus *Pratylenchus*. In this study, *P. coffeae* was confirmed as a pathogen causing root rot in sorghum by Koch’s law tests. *P. coffeae* infests the root system of sorghum, causing cell necrosis, resulting in root rot and other symptoms. When the density of the nematode population is high, plant dwarfing can occur, and the sculpts leaves show signs of nutrient deficiency. In severe cases, the aboveground part of the plant fades to green, grows slowly, yellows, and even dies [5, 7].

The parasitism and pathogenicity of the *P. coffeae* GL-1 population and four other populations on sorghum were determined via greenhouse pot inoculation tests in our study. The host status and susceptibility of sorghum to four root-lesion nematodes were studied in greenhouse and field microplot experiments [10]. Sorghum was a good host for *P. zeae*, *P. brachyurus* and *P. coffeae* which had reproduction indices (Pf/Pi) greater than 1, Sorghum was a nonhost for *P. crenatus*. Our findings are consistent with the findings of Motalaote’s study, all five populations of *P. coffeae* from different host sources were able to cause root pathogenicity in sorghum, but there were significant differences in the pathogenicity of *P. coffeae* in sorghum from different regions or host sources. These differences may be due to environmental [27], anthropogenic [28], and intrinsic nematode factors [29], which are common to a wide range of root-lesion nematodes. Nematodes are adapted to and tamed over time in different environments, and humidity and temperature alter their reproductive and pathogenic capacities [30].

In agriculture, sorghum is often rotated or intercropped with crops such as soybeans, corn, or wheat to achieve greater yields [2]. Soybean and corn, among other crops, are suitable hosts of *P. coffeae* [31, 32]. This study demonstrated that *P. coffeae* isolated from the inter-root of soybeans and corn can infest sorghum roots. We hypothesize that *P. coffeae* is capable of infesting successive interspecific crops in the soil if all crops grown in a rotation or sets of crops are hosts of *P. coffeae*. In our study, this is the first time that *P. coffeae* can cause significant damage to sorghum in the field and it should continue to be a concern in sorghum cultivation in the future.

## Conclusions

In this study, the pathogen causing sorghum root rot disease found in Shanxi Province, China, was identified as *P. coffeae*, and this nematode population and four other populations of *P. coffeae* conserved in our laboratory were subsequently subjected to a pot inoculation test to determine their parasitism and pathogenicity on sorghum plants. The indoor potting results demonstrated that sorghum is a suitable host for *P. coffeae* (Rf > 1) and that all five different populations of *P. coffeae* can grow, develop, and complete their life cycles within the sorghum root system. All five different test *P. coffeae* populations were highly pathogenic to sorghum, but there were significant differences in pathogenicity between populations with different hosts or from different geographic origins. To our knowledge, this is the first report of root rot in *Sorghum bicolor*, caused by *P. coffeae* in China. This study provides a scientific basis for identifying and detecting root-lesion nematodes in sorghum.

## Electronic supplementary material

Below is the link to the electronic supplementary material.

Supplementary Material 1

Supplementary Material 2

## Data Availability

The datasets generated and analysed during the current study are available in the NCBI repository or in Annex 1 and 2 with the accession codes PP531525 (ITS) (https://www.ncbi.nlm.nih.gov/nuccore/PP531525.1/) and PP531576 (28 S) (https://www.ncbi.nlm.nih.gov/nuccore/PP531576.1/). The authors confirm that all data underlying the findings are fully available without restriction. All relevant data are within the paper.
